# Innate Sensing of HIV-1 Assembly by Tetherin Induces NFκB-Dependent Proinflammatory Responses

**DOI:** 10.1016/j.chom.2012.10.007

**Published:** 2012-11-15

**Authors:** Rui Pedro Galão, Anna Le Tortorec, Suzanne Pickering, Tonya Kueck, Stuart J.D. Neil

**Affiliations:** 1 Department of Infectious Disease, King’s College London School of Medicine, Guy’s Hospital, London SE1 9RT, UK

## Abstract

Antiviral proteins that recognize pathogen-specific or aberrantly located molecular motifs are perfectly positioned to act as pattern-recognition receptors and signal to the immune system. Here we investigated whether the interferon-induced viral restriction factor tetherin (CD317/BST2), which is known to inhibit HIV-1 particle release by physically tethering virions to the cell surface, has such a signaling role. We find that upon restriction of Vpu-defective HIV-1, tetherin acts as a virus sensor to induce NFκB-dependent proinflammatory gene expression. Signaling requires both tetherin’s extracellular domain involved in virion retention and determinants in the cytoplasmic tail, including an endocytic motif, although signaling is independent of virion endocytosis. Furthermore, recruitment of the TNF-receptor-associated factor TRAF6 and activation of the mitogen-activated protein kinase TAK1 are critical for signaling. Human tetherin’s ability to mediate efficient signaling may have arisen as a result of a five amino acid deletion that occurred in hominids after their divergence from chimpanzees.

## Highlights

► Restriction of HIV-1 release by human tetherin induces NFκB-dependent gene expression ► Tetherin-sensitive HIV-1 mutants induce enhanced proinflammatory cytokine expression ► Signaling requires tetherin’s extracellular domain and determinants in the cytoplasmic tail ► TAK1 activation and TRAF6 recruitment are critical for tetherin-dependent signaling

## Introduction

Tetherin is a broadly acting antiviral membrane protein that blocks the release of diverse mammalian viruses from the surface of infected cells and reduces retroviral pathogenesis in vivo (Liberatore and Bieniasz, 2011; Martin-Serrano and Neil, 2011). Tetherin partitions into assembling virions, crosslinking them to the plasma membrane (PM) by virtue of its unique topology. The potential importance of tetherin in innate antiviral immunity is underscored by examples of virally encoded proteins that counteract its activity, the prototype being the accessory gene product Vpu of human immunodeficiency virus type 1 (HIV-1) (Le Tortorec et al., 2011). Vpu interacts with human tetherin in infected cells, blocking its transit to viral assembly sites on the PM (Dubé et al., 2011; Kueck and Neil, 2012; Schmidt et al., 2011) and promoting its endosomal degradation (Agromayor et al., 2012; Janvier et al., 2011). Among the primate lentiviruses, the species-specific targeting of tetherin is widespread, even though the *vpu* gene is restricted to a subset of simian immunodeficiency viruses (SIVs). In SIVs in which Vpu is absent, the Nef accessory protein performs the role, and the sensitivity of primate tetherins to SIV Nefs is determined by a five amino acid patch in the protein’s cytoplasmic tail that was deleted after hominids and chimpanzees diverged (Jia et al., 2009; Zhang et al., 2009).

Accumulating evidence indicates that tetherin imposes a powerful selective pressure on primate lentiviruses and that its counteraction is essential for HIV/SIV replication and spread in vivo. Adaptation of the SIVcpz Vpu to target human tetherin efficiently is a feature of the major group (Group M) of HIV-1 that is responsible for the HIV/AIDS pandemic, but not the separate SIVcpz species jumps that led to the N, O, and P groups that have remained geographically restricted (Sauter et al., 2009). Zoonotic spread of the Vpu(−) SIV of sooty mangabeys (SIVsm) to become HIV-2, whose Nef protein cannot target human tetherin, resulted in tetherin counteraction developing in the envelope protein (Env) (Le Tortorec and Neil, 2009). Furthermore, a similar adaptation in Env has been observed in macaques experimentally infected with a Nef-defective SIVmac that reverted to pathogencity (Serra-Moreno et al., 2011).

While tetherin potently blocks cell-free virion release, its ability to block cell-to-cell transfer is controversial, and where it has been observed, weak (Casartelli et al., 2010; Jolly et al., 2010). In cultured primary CD4+ T cells, Vpu-defective viruses have even been observed to spread faster through the culture in a tetherin-dependent manner (Jolly et al., 2010). These superficially paradoxical observations between viral replication in culture and the evolutionary conservation of tetherin counteraction in primate lentiviruses suggest that tetherin’s contribution to the antiviral immune response is not limited to physical inhibition of virion release. Interestingly, prior to its identification as an antiviral factor, tetherin (BST2) was identified as an inducer of NFκB activation in a whole-genome transfection screen (Matsuda et al., 2003). Furthermore, tetherin has been implicated as a regulator of Toll-like receptor (TLR) function in plasmacytoid dendritic cells through the activation of the inhibitory leukocyte receptor ILT7 (Cao et al., 2009). We therefore hypothesized that tetherin, like the retroviral restriction factor TRIM5α, might act as a sensor for the presence of viral infection coupled to its antiviral activity (Pertel et al., 2011). In this study we tested this hypothesis.

## Results

### Restriction of Enveloped Virus Particle Release by Human Tetherin Induces NFκB-Dependent Gene Expression

**Figure 1:**
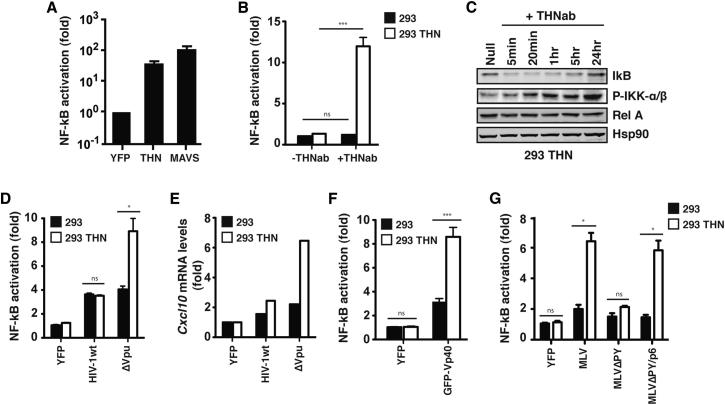
Tetherin Induces NFκB-Dependent Responses upon Overexpression, Crosslinking, and Restriction of Virion Release (A) Fold activation of a firefly-luciferase NFκB reporter gene in 293 cells transiently cotransfected with tetherin, MAVS, or control YFP vectors. (B) Fold activation of the same reporter in 293 or 293THN cells treated for 24 hr with a rabbit anti-tetherin polyclonal serum and a secondary anti-rabbit antibody. (C) Time course of endogenous IκB degradation and IKKα/β phosphorylation in 293THN cells after antibody crosslinking. (D and E) (D) Fold increases in NFκB-reporter activity in 293 and 293THN cells transfected with wild-type and Vpu(−) HIV-1 proviruses and (E) fold changes in *Cxcl10* mRNA levels compared to YFP transfection calculated relative to *Gapdh* by qRT-PCR. (F and G) NFκB-reporter fold activation in 293 and 293THN cells transfected with GFP-fused Ebolavirus VP40 expression vector (F) or MLV provirus or derivatives (MLVΔPY and MLVΔPY/p6) (G). Fold changes relative to 293 cells transfected with YFP control (A, D–G) or 293 nontreated cells (B). ^∗^p > 0.05 and ^∗∗∗^p > 0.001 as determined by two-tailed t test. All error bars represent ±SEM of three independent experiments.

We first sought to confirm whether tetherin was capable of inducing NFκB activation when overexpressed. Transient transfection of human tetherin into 293 cells potently induced the activation of an NFκB-dependent firefly luciferase reporter construct to levels similar to those seen with the MAVS/IPS1/Cardif component of the cytoplasmic viral RNA-sensing pathway (Takeuchi and Akira, 2010), confirming the previous observation from the genomic screen (Matsuda et al., 2003) (Figure 1A). We then examined whether crosslinking of cell-surface tetherin was capable of transducing a similar signal in 293 cells stably expressing human tetherin (293THN), but not vector control 293 cells. While background level of reporter gene activation in the two cell types was less than 1.5-fold, crosslinking the surface protein with a polyclonal antibody resulted in a 10-fold increase in NFκB-dependent luciferase expression in tetherin-expressing cells (Figure 1B) and led to the concomitant phosphorylation of IKKα/β and degradation of IκB (Figure 1C). This suggested that clustering of tetherin might mediate a proinflammatory signal upon restriction of enveloped virus release. Consistent with this, budding and assembly of HIV-1 lacking the tetherin countermeasure Vpu (Neil et al., 2008), or encoding a point mutant of Vpu (A14L) that is defective for interaction with tetherin (Vigan and Neil, 2010), specifically induced enhanced NFκB reporter activation in cells stably expressing tetherin while the wild-type virus did not, correlating with physical particle release (Figure 1D and see Figures S1A and S1B online). Furthermore, consistent with the activation of the reporter gene, we could observe induction of the mRNA of a representative NFκB-dependent target gene, the proinflammatory chemokine CXCL10, in the same cells (Figure 1E).

Since tetherin restricts the release of a variety of mammalian viruses, we then sought to determine whether other tetherin-sensitive viral particles could activate the NFκB reporter. We observed similar reporter activation by producing tetherin-sensitive filoviral-like particles (VLPs) derived from the Ebolavirus matrix protein VP40 (Jouvenet et al., 2009; Neil et al., 2007) (Figure 1F and Figure S1C). Furthermore, since previous studies have demonstrated that tetherin restricts the release of fully assembled virions that have separated their membranes from that of the host cell, we then examined whether assembly-defective retroviral particles could trigger tetherin-dependent NFκB activation. For this we used a murine leukemia virus (MLV) provirus, or derivatives with mutations in the p12^Gag^ late-domain sequence (MLVΔPY) that facilitates virion budding and membrane scission by recruiting the ESCRT-pathway (Martin-Serrano and Neil, 2011). As expected, wild-type MLV release was restricted in 293THN cells, whereas MLVΔPY release was defective in both cell types (Figure S1D). Production of wild-type MLV virions triggered enhanced NFκB reporter activity in 293THN cells, but the assembly-defective MLVΔPY did not (Figure 1G). Interestingly, MLVΔPY/p6, where the p12 late domain has been replaced with one derived from HIV-1 p6^gag^, recovered both virion production and the enhanced NFκB reporter activation in 293THN cells (Figure 1G and Figure S1D). Therefore activation of NFκB in tetherin-expressing cells requires full virion assembly and scission of the cellular and virion membranes. Together these data show that in cells constitutively expressing tetherin, the restriction of enveloped virion release triggers a proinflammatory response.

### Tetherin-Sensitive HIV-1 Mutants Induce Enhanced Proinflammatory Cytokine Expression in Infected CD4+ T Cells

**Figure 2:**
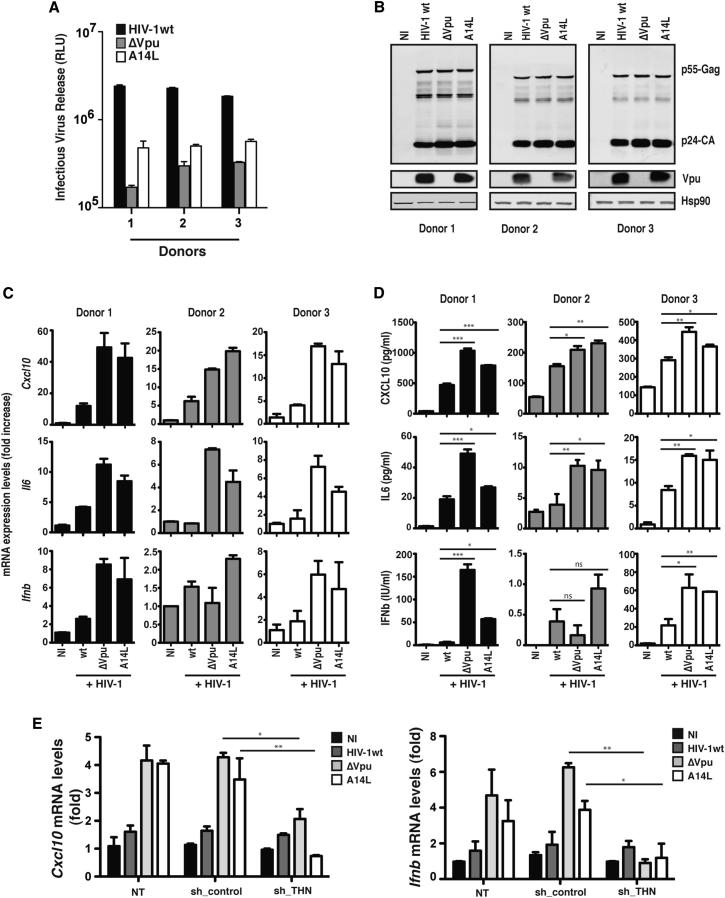
Restriction of Tetherin-Sensitive HIV-1 Mutant Release from Infected CD4+ T Cells Induces Proinflammatory Gene Expression (A and B) Purified CD4+ T cells from three independent donors were infected with the indicated virus at an moi of 5, and 48 hr later viral release was determined by infection of HeLa-TZM reporter cell lines (A) and western blot of cell lysates (B). (C) Total RNA from three biological replicates of parallel cultures infected as in (A) was analyzed for *Cxcl10*, *IL6*, and *Ifnb* mRNA levels relative to *Gapdh* by qRT-PCR. (D) Protein levels in the supernatants from (A) were determined by ELISA (CXCL10 and IL-6) or by HEK-Blue indicator cells (IFNβ). (E) Activated CD4+ T cells were transduced with lentiviral vectors encoding short hairpin against GFP (sh-control) or against tetherin (shTHN) 24 hr prior to infection as in (A). Total RNA from three biological replicates of parallel cultures was analyzed for *Cxcl10* and *Ifnb* mRNA levels relative to *Gapdh* by qRT-PCR. Fold changes relative to noninfected cells (C and E). ^∗^p > 0.05, ^∗∗^p > 0.01, and ^∗∗∗^p > 0.001 as determined by two-tailed t test. All error bars represent ±SEM.

We then asked whether tetherin-mediated restriction of HIV-1 particle release in primary human CD4+ T cells would induce proinflammatory gene expression. Purified CD4+ T cells from three donors were infected with wild-type HIV-1, Vpu(−) HIV-1, or HIV-1 encoding tetherin binding-defective mutant Vpu-A14L that retains all other known Vpu functions. As expected, cell-free virus release of the Vpu mutant HIV-1s was impaired compared to wild-type in all donors (Figures 2A and 2B). Interestingly, cultures infected with both Vpu(−) and Vpu-A14L HIV-1 mutants led to increased levels of mRNAs for proinflammatory cytokines CXCL10 and IL-6 (Figure 2C). In two of the donors, increased levels of *Ifnb* mRNA were also detected (Figure 2C). Furthermore, these increases in mRNA levels were reflected in significant increases in the concentrations of CXCL10, IL-6, and bioactive type-1 IFN in the supernatant (Figure 2D). The induction of type 1 IFN expression implied the activation of interferon regulatory factors (IRFs) 3 or 7 in addition to NFκB. In parallel reporter gene experiments to those described above, however, tetherin overexpression, crosslinking, or Vpu(−) HIV-1 assembly could not activate an IFNβ-promoter-luciferase reporter construct in 293THN cells. Neither did we observe any induction of *ifnb* mRNA or phosphorylation of IRF3 in this system (Figures S2A–S2C). However, in CD4+ T cells we could detect IRF3 phosphorylation after 48 hr of HIV-1 infection. In contrast to the induction of the NFκB-dependent *Cxcl10* mRNA in T cells or tetherin-mediated reporter gene activation in 293THN cells, this IRF3 phosphorylation and the concomitant IFNβ expression could be suppressed by inhibition of the TLR3 adaptor TRIF (Figures S2D–S2G), suggesting that tetherin-mediated viral retention may additionally augment viral recognition by other host PRRs. Therefore, to confirm the tetherin dependency of all these data, we transduced primary CD4+ T cells with lentiviral vectors encoding shRNA hairpins against tetherin (shTHN) or an irrelevant target (GFP) 24 hr prior to infection with the above viruses. As expected, shTHN transduction reduced surface tetherin levels and rescued the release of Vpu(−) and Vpu-A14L mutant viruses to wild-type levels (Figures S2H and S2I). It also concomitantly reduced both *Cxc10* and *Ifnb* mRNA induction in the same cells (Figure 2E), demonstrating that proinflammatory gene expression induced by Vpu(−) HIV-1 in target cells is tetherin dependent. We thus provide evidence that interaction of tetherin with HIV-1 induces proinflammatory gene expression consistent with a direct signaling role.

### Structural Determinants of Tetherin-Mediated Signaling

**Figure 3:**
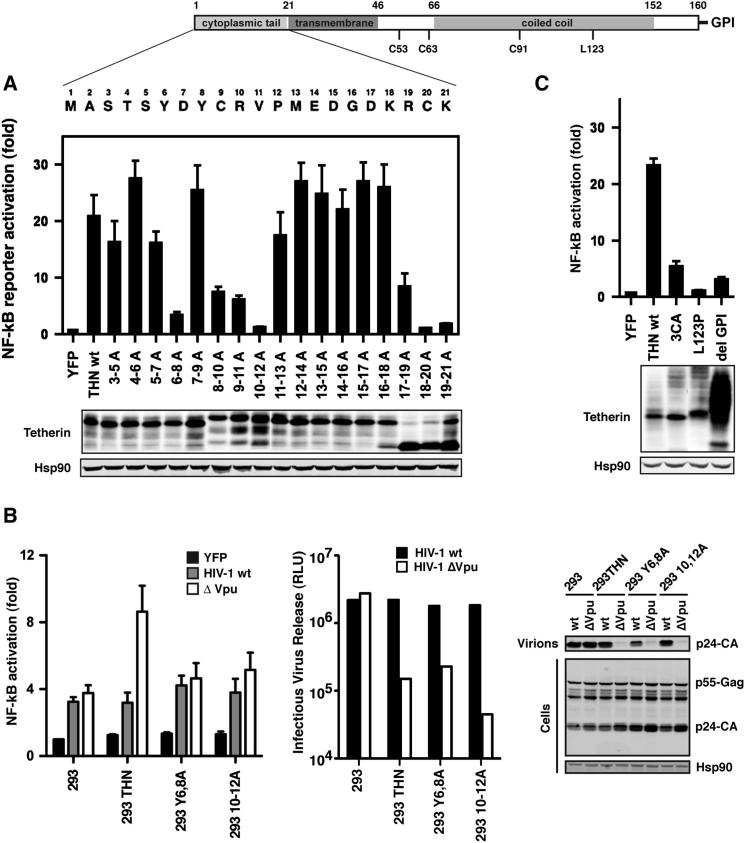
Determinants of Tetherin-Mediated Signaling (A) A series of alanine scan mutants in the cytoplasmic tail of human tetherin were assessed for the ability to induce NFκB-Luc reporter activation in transiently transfected 293 cells. Mutations encompassing residues 17–21 of the cytoplasmic tail were discounted due to aberrant localization (data not shown), and further mutants are presented in Figure S3. Error bars are ±SEM. (B) 293 cells stably expressing the Y6,8A and 10-12A mutants were further assessed for their ability to induce NFκB-Luc reporter activation upon transfection with wild-type or Vpu(−) HIV-1 proviral plasmids. Infectious virus release was determined on HeLa-TZM indicator cells and physical particle yield analyzed by western blot of cell lysates and pelleted supernatants using an anti-p24 monoclonal antibody. (C) Mutations in the extracellular domain of tetherin defective for inhibiting viral release (cysteine-less 3CA, the coiled-coil point mutant L123P, or a truncation lacking the GPI anchor) were assayed for NFκB-Luc reporter activation in transfected 293 cells. Fold changes ±SEM relative to 293 cells transfected with YFP control (B and C).

We then mapped the determinants of NFκB activation in human tetherin. The primary sequence of the tetherin cytoplasmic tail does not affect the protein’s antiviral activity (data not shown). However, we reasoned that the cytoplasmic tail likely played a role in signaling and screened a series of tetherin cytoplasmic tail mutants for NFκB-dependent reporter gene activation (Figure 3A). This revealed defects in signaling that mapped to the conserved YXY site that mediates tetherin’s endocytic recycling to the PM (Rollason et al., 2007), as well as an adjacent CRV motif. Mutations encompassing the two membrane-proximal lysine residues, which can serve as ubiquitylation targets (Le Tortorec et al., 2011), were also defective for signaling. However, these alanine mutants appeared immature in SDS-PAGE, suggesting a trafficking defect, and mutation of the individual lysine residues to arginine preserved signaling capacity (Figure S3A). Stable cell lines expressing tetherin Y6,8A and 10-12A failed to mediate reporter-gene activation in response to Vpu(−) HIV-1, yet they retained potent viral particle restriction, consistent with the hypothesis that these motifs are required for transducing a signal initiated by virion retention (Figures 3B). By contrast, the antiviral activity of tetherin depends upon the structural integrity of its extracellular domain, particularly in its C-terminal GPI-linked membrane anchor, its dimeric state, and its extracellular coiled-coil domain (Perez-Caballero et al., 2009). Mutations in the coiled coil (L123P), the extracellular cysteine residues that mediate dimerization, or deletion of the GPI anchor, all of which render tetherin unable to inhibit viral release, reduced the ability of tetherin to promote NFκB-reporter gene activation both in transient transfections and in stably expressing cells transfected with tetherin-sensitive and -insensitive HIV-1 (Figure 3C and Figure S3B). Thus tetherin’s ability to mediate NFκB activation requires the extracellular determinants essential for multimerization and virion retention, and cytoplasmic tail motifs required to transduce a signal dependent on this retention.

### Tetherin-Mediated Signaling Is Independent of Virion Endocytosis

**Figure 4:**
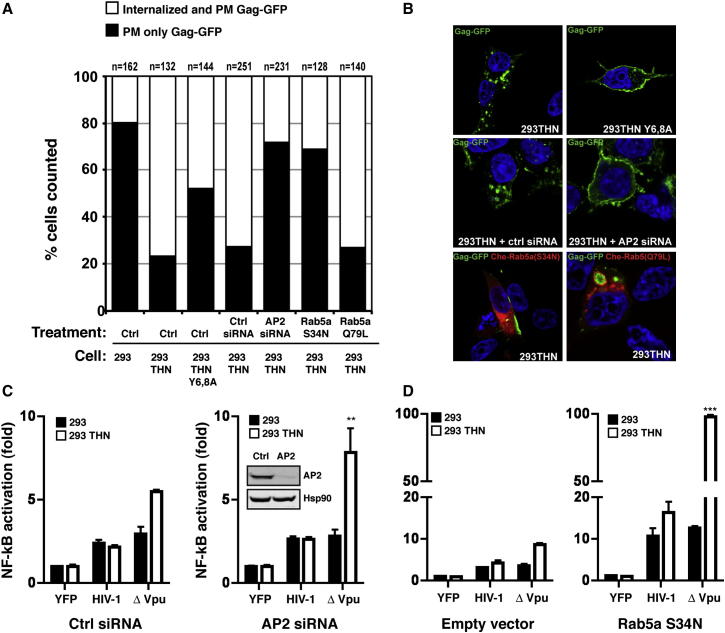
Inhibition of Virion Endocytosis Enhances Tetherin-Dependent NFκB Activation (A and B) The indicated cell lines were transfected with an HIV-1 Gag-GFP expression vector, with or without either pretreatment with control siRNA or AP2μ1 for 48 hr or cotransfection with Cherry-FP fused mutants of Rab5a (S34N or Q79L). Twenty-four hours later, the cells were fixed and ten random fields were enumerated on the basis of whether transfected cells displayed plasma-membrane only Gag-GFP localization or PM and distinct endsomal accumulation (A) and imaged by confocal microscopy (B). (C and D) The effects of AP2μ1 siRNAs and Che-Rab5a-S34N on HIV-1 proviral-induced NFκB reporter gene activation were determined as above. Fold changes relative to 293 cells transfected with YFP control with error bars representing ±SEM.

Tetherin-mediated retention of Vpu(−) HIV-1 virions leads to their accumulation in late endosomal compartments (Martin-Serrano and Neil, 2011). The implication of the YXY motif, which has been previously shown to interact with both AP1 and AP2 clathrin adaptors, suggested that endocytosis of virions might be required for signaling. Consistent with this, a lower proportion of cells exhibited distinct endosomal accumulations of HIV-1 Gag-GFP in 293THN-Y6,8A compared to 293THN 24 hr posttransfection (48% versus 74%), suggesting that the YXY motif may play a role in virion uptake (Figures 4A and 4B). Recent evidence suggests that YXY mutants of tetherin are impaired but not fully defective for endocytosis (Lau et al., 2011), potentially accounting for this partial phenotype. However, siRNA depletion of AP2μ1 reduced endosomal virion localization to levels comparable to the parental 293 cells, similar to the expected result observed by cotransfecting a dominant inhibitory mutant S34N of the early endocytic GTPase Rab5a (Neil et al., 2008). As a control, the proportion of transfected cells with observable endosomal Gag-GFP was unchanged when using the dominantly active mutant, Rab5a(Q79L), with Gag-GFP VLPs accumulating in the lumen of swollen Rab5+ve endosomes. We then examined the effects of these treatments on virion-induced NFκB-reporter activation in 293THN cells. As expected, Vpu(−) HIV-1 induced an increase in reporter activation compared to wild-type virus. However, in the presence of AP2μ1 depletion or Rab5a(S34N) overexpression, Vpu(−) virus-induced signaling was significantly increased in 293THN cells (Figures 4C and 4D). These data suggest that while the YDYCRV motif is essential for virion-induced signaling, virion uptake is not, and blockade of their delivery to late endosomes potentiates reporter gene expression. Together, they suggest that tetherin-dependent accumulation of virions on the cell surface triggers NFκB activation.

### TAK1 Activation and Recruitment of TRAF6 Are Required for Tetherin-Mediated Signaling

**Figure 5:**
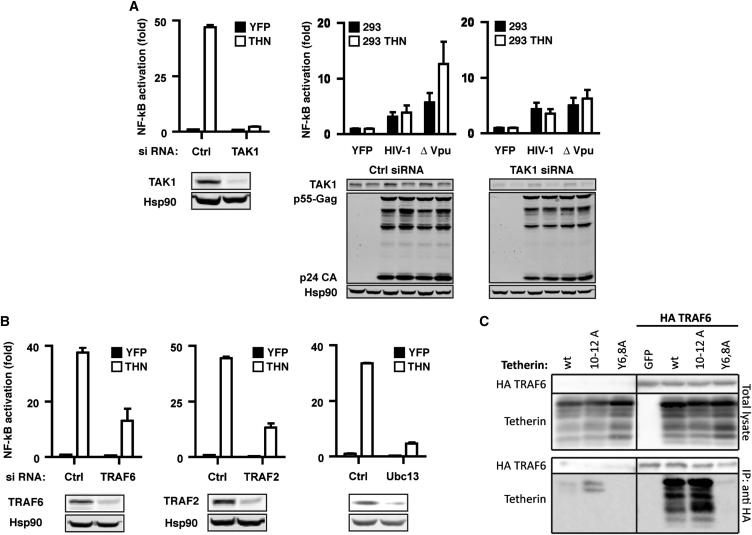
Mechanistic Aspects of Tetherin-Mediated Signaling (A) Depletion of TAK1 by siRNA in either 293 cells transfected with tetherin, or in 293 and 293THN cells transfected with Vpu(−) HIV-1 proviral plasmid, abolishes NFκB-Luc reporter activation but has no effect on Gag production. (B) Impairment of NFκB-Luc reporter activation by siRNA depletion of TRAF6, TRAF2, or Ubc13 in 293 cells transfected with tetherin. (C) Coimmunoprecipitation of tetherin and tetherin(10-12A) but not tetherin Y6,8A with HA-TRAF6 in transiently transfected 293 cells. All error bars are ±SEM of three independent experiments.

Proinflammatory activation of NFκB from TNF receptors and membrane-associated and cytosolic PRRs is mediated through the mitogen-activated protein kinase TAK1 (MAP3K7) (Skaug et al., 2009). We therefore asked whether there were similarities between tetherin-mediated NFκB activation and that of other viral PRRs. Depletion of TAK1 by siRNA completely abolished the NFκB-reporter gene activation induced by tetherin overexpression or Vpu(−) HIV-1 in 293THN cells (Figure 5A), implicating the activation of TAK1 as critical in tetherin-mediated signaling. TAK1 activation by host PRRs depends on the upstream recruitment of family members of the TNF-receptor-associated factor (TRAF) family of E3 ubiquitin ligases, particularly TRAF2 and TRAF6, and the E2 ligase Ubc13 (UBE2N), which act to polymerize K63-linked ubiquitin chains (Skaug et al., 2009). RNAi depletion of these factors similarly inhibited tetherin-mediated NFκB reporter activity (Figure 5B). Interestingly, while the human tetherin cytoplasmic tail has a putative TRAF6 consensus-binding motif (PXEXX-aromatic/acidic), disruption of this sequence by mutagenesis did not impair tetherin signaling (Figure 3). However, tetherin could be specifically coimmunoprecipitated with an HA-tagged TRAF6 dependent on the presence of the YCRV motif that is conserved in ape tetherins and partially overlaps the protein’s endocytic motif (Figure 5C and Figure S4), although whether this interaction is direct is unknown. Furthermore, mutation of residues 10–12 (RVP) retained TRAF6 interaction despite losing signaling activity, indicating that in conjunction with the RNAi data, TRAF6 is necessary but not sufficient for tetherin signaling. These data suggest that virion aggregation by tetherin leads to YDYCRV-dependent recruitment of a signaling complex that includes TRAF6 to induce TAK1-dependent NFκB activation when viral release is restricted.

### Signaling Is a Feature of Human and Chimpanzee Tetherins and Is Augmented by a Hominid-Specific Deletion in the Tetherin Cytoplasmic Tail

**Figure 6:**
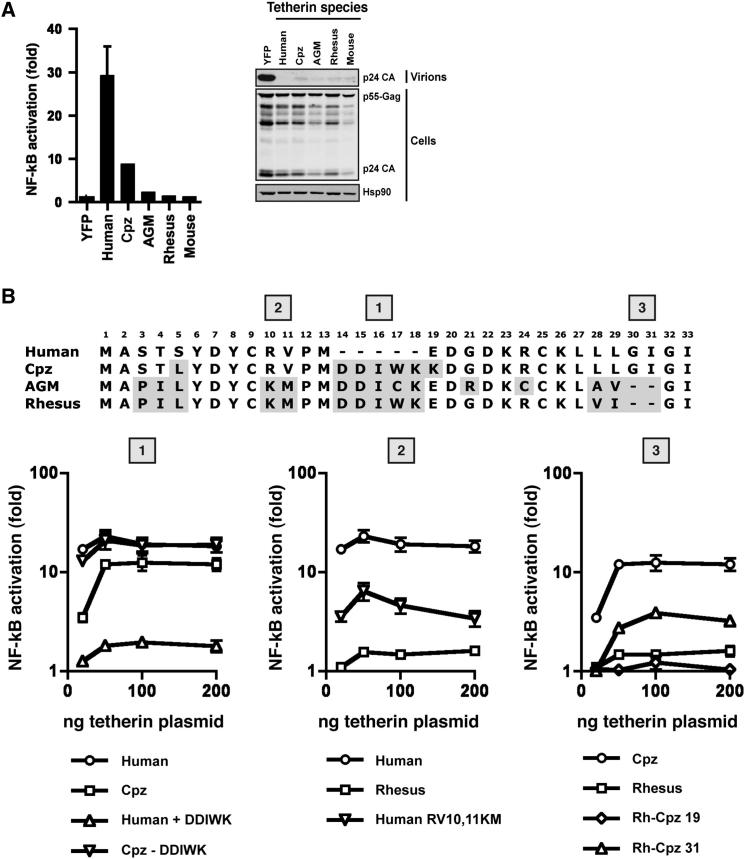
Tetherin’s Signaling Activity Is a Recent Acquisition in Primate Evolution (A) NFκB-Luc reporter activation in 293 cells transiently transfected with murine, rhesus, AGM, chimpanzee, and human tetherins and their respective ability to restrict Vpu-defective virus release. (B) Alignment of the cytoplasmic tails of primate tetherins with regions differing from human shown in gray. Species-specific changes were made according to major differences indicated at positions 1, 2, and 3 and tested for NFκB-Luc reporter activation at varying expression levels. All error bars represent ±SEM of three independent experiments.

Tetherin’s antiviral activity is conserved in mammals. Surprisingly, however, murine and old world monkey tetherins showed negligible ability to induce NFκB reporter activity despite potently restricting HIV-1 particle release (Figure 6A). Chimpanzee tetherin (cpz-tetherin), while capable of inducing reporter gene activation, was less potent than the human protein (Figure 6A). Informed by our mutagenesis studies (Figure 3), we identified three regions of species-specific changes between the cytoplasmic tails of human, chimpanzee, and rhesus tetherins that might contribute to the differences in signaling. First, a major difference between human and other primate proteins is the presence of a G/DDIWK motif that is absent in *Homo sapiens*. The tryptophan in this motif is under high evolutionary positive selection and determines the sensitivity of primate tetherins to the Nef proteins of SIVs (Lim et al., 2010). Second, while the two tyrosine residues in the YXYXXV endocytic motif are highly conserved throughout mammalian evolution, differences occur between humans/chimpanzees and old world monkeys at positions 9–11, a region we have identified as critical to human tetherin’s signaling ability. Both the C9 (Gupta et al., 2009; Lim et al., 2010) and R10 (Gupta et al., 2009; McNatt et al., 2009) positions have been previously ascribed to be under positive selection throughout primate evolution. Third, in human and chimpanzee tetherins a repeated glycine-isoleucine pair extends the tetherin transmembrane helix, and is an important determinant of sensitivity to HIV-1 Vpu (McNatt et al., 2009). A further difference between human, chimpanzee, and rhesus encompasses an ubiquitination site (STS) (Tokarev et al., 2011), although our alanine mutagenesis suggests that this motif is not important.

Transfection of increasing amounts of hu- and cpz-tetherin plasmids revealed that at low expression levels human tetherin was markedly more efficient at inducing NFκB-reporter activation, and high levels of the chimpanzee protein never achieved parity (Figure 6B). Deletion of the DDIWK motif enhanced cpz-tetherin-mediated NFκB reporter gene activation to the level of human protein, and its reintroduction into the human protein severely impaired signaling. Neither of these changes affected either expression, restriction of Vpu(−) HIV-1 release, or TRAF6 association (Figure 6B and Figure S5). Therefore efficiency of NFκB activation between hu-tetherin and cpz-tetherin appears to be an evolutionarily recent acquisition that has coincided with the deletion of a motif known to act as a target for SIV tetherin countermeasures.

We then turned our attention to rh-tetherin. In rhesus macaques, position 9 is polymorphic (either a C or an R), and neither variant could mediate NFκB-reporter activity. Similarly, a C9R change in human tetherin had no effect on signaling ability (data not shown). Introduction of KM-to-RV changes into rh-tetherin did not endow it with any signaling activity, but the corresponding RV-to-KM swap in human tetherin severely reduced its NFκB-reporter induction without compromising antiviral activity, confirming that these residues are necessary in human, but not sufficient to explain the lack of rhesus signaling (Figure 6B). To determine the minimal changes in rh-tetherin that could confer signaling activity, we constructed chimeras between rh-tetherin and cpz-tetherin. Again all proteins were capable of restricting virion release. Surprisingly, only a chimera bearing the cpz-tetherin cytoplasmic tail including the additional TM-domain GI residues (rh-cpz 31) recovered any NFκB activation (Figure 6B). These results suggest multiple context-dependent adaptations have resulted in human tetherin’s signaling capacity. First, changes in the previously identified YXYXXV motif in conjunction with a lengthened TM domain, which itself may be expected to alter the conformation of the cytoplasmic tail, are required to confer levels of signaling activity seen in cpz-tetherin. Second, deletion of the DDIWK motif in human ancestors greatly enhanced the efficiency of this activity.

## Discussion

In this study we have demonstrated that human tetherin can transduce a proinflammatory signal when it restricts virion production. This appears to be coupled to cell-surface aggregation of virions, resulting in the TAK1-dependent activation of NFκB and subsequent cytokine production. Importantly, we could show that tetherin-sensitive mutants of HIV-1 induce enhanced proinflammatory cytokine production in infected CD4+ T cells. This suggests that the action of tetherin senses enveloped virus assembly, and that the infected cell responds by releasing mediators that can attract innate and adaptive immune cells to the site of infection to augment the antiviral response. Thus we suggest that in the context of an infected cell, a budding enveloped virion constitutes a pathogen-associated molecular pattern, and in addition to physically restricting virion release, tetherin acts as the cognate PRR.

Mechanistically, tetherin signaling has similarities to TLRs and other viral PRRs, including another retroviral restriction factor, TRIM5α (Pertel et al., 2011; Skaug et al., 2009; Takeuchi and Akira, 2010). NFκB is activated through the upstream kinase TAK1. PRR-mediated TAK1 activation depends on the association of its accessory factors TABs 1–3 with free polyubiquitin chains bearing K63 linkages, which in turn are synthesized by the concerted action of TRAF E3 ligases with E2 complex of Ubc13/UBE1. TRAFs, particularly TRAFs 2, 3, 5, and 6, play key roles in signal transduction from a host of proinflammatory cell-surface receptors, and TRAF2 and TRAF6 are also essential cofactors for cytoplasmic RIG-I-like helicases. We found that RNAi-mediated knockdown of TRAF2, TRAF6, and Ubc13 inhibited tetherin activation of NFκB, for which TAK1 was also essential. Furthermore we could observe an interaction between tetherin and TRAF6. While the human tetherin cytoplasmic tail contains a predicted TRAF6 binding site, we found that this interaction was actually determined by the adjacent endocytic motif YDYCRV. How the YDYCRV motif recruits TRAF6, and how this relates to its endocytic function, remains to be determined. While signaling is dependent on this signal, which appears also to play a partial role in virion internalization, blockade of virion uptake itself by AP2μ siRNAs or Rab5(S34N) potentiates NFκB activation. Since these treatments do not affect tetherin’s physical antiviral activity per se, these data strongly suggest that large surface virion accumulations are the primary trigger of tetherin-mediated signaling.

TRAF6 recruitment is not sufficient to explain tetherin-mediated signal transduction, as mutation of residues 10–12 (RVP) or reinsertion of the primate DDIWK motif also impairs signaling while retaining TRAF6 binding ability. Interestingly, both the cysteine (Lim et al., 2010) and the arginine (Gupta et al., 2009; McNatt et al., 2009) residues have been reported to be under positive selection in primates, and the YCRV motif is only found in great apes. While the cysteine appears not to be required, mutation of the R and V to the K and M found in macaques impaired human tetherin signaling. Furthermore, the context dependency on the insertion of the glycine-isoluecine pair in the TM domain that allows gain of function of the rh-tetherin when fused to the cpz-tetherin tail suggests that a global structural change in the orientation of the tetherin cytoplasmic tail was a prerequisite for the acquisition of signaling capacity. One caveat to the interpretation of these observations is that at present they have not been performed in the cells from their cognate primate species. Thus it is unknown whether any of the context dependency reflects species-specific differences in downstream cellular partners. Also whether similar signaling attributes have developed in mammalian tetherins other than those tested here remains to be determined.

**Figure 7:**
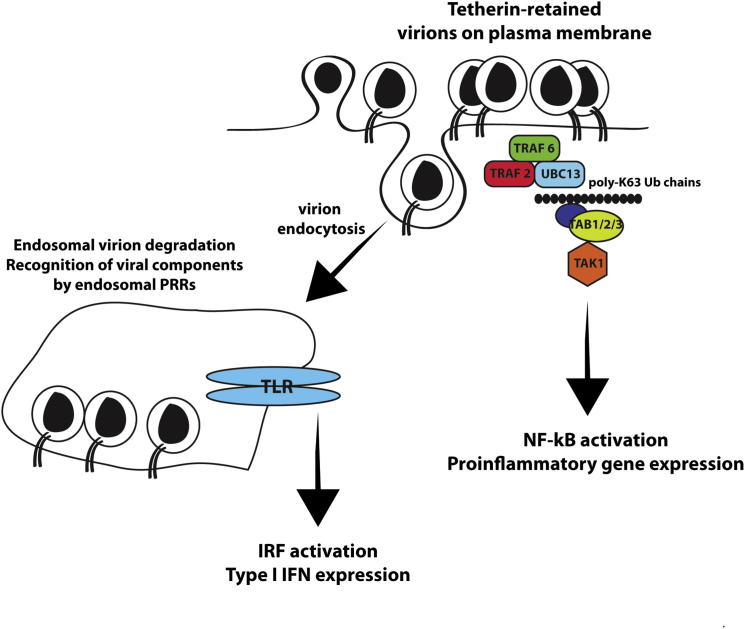
Model for Tetherin’s Proinflammatory Activity Clustering of tetherin dimers upon restriction of viral release promotes the recruitment of a signaling complex that includes TRAF6, and potentially TRAF2 and Ubc13 dependent on the YDYCRV motif in tetherin. This leads to the activation of TAK1, NFκB, and enhanced proinflammatory gene expression by the infected cells. Additionally, in primary HIV-1 target cells, virion retention and uptake potentially targets restricted virions to compartments where viral components are amenable to recognition by TLRs or other PRRs, thereby indirectly inducing type 1 IFN responses through the activation of IRFs.

We propose the following model (Figure 7): tetherin-mediated virion retention triggers clustering of surface tetherin, targeting it for endocytic uptake. Coupled to this surface clustering, a signaling complex containing TRAF6 and perhaps TRAF2 is recruited to the tetherin cytoplasmic tail. What other factors are present in this complex, or whether it directly interacts with tetherin itself or an associated factor, remains to be determined. The recruited complex then activates TAK1 through the polymerization of ubiquitin chains dependent on Ubc13, which in turn leads to IKK phosphorylation, IκB degradation, and translocation of NFκB to the nucleus to activate proinflammatory gene expression. The data presented herein showing that late-domain mutants of retroviral particles cannot trigger tetherin signaling indicate that partitioning of tetherin into virions initiates signaling dependent on the physical separation of budding virions from the cell membrane. The topology of tetherin dimers that form the physical tether is not entirely clear (Perez-Caballero et al., 2009), but the evidence that they can transduce a signal implies that at least some tetherin dimers must remain with their cytoplasmic tails in the cytoplasm. Recently it was demonstrated that HIV-1 gp41 can also activate TAK1-dependent NFκB signaling to potentiate viral replication in CD4+ T cells (Postler and Desrosiers, 2012). Whether this underlies the lower level of responses that we see in wild-type infected cells is unknown, but may suggest that HIV-1 strikes a balance between maintaining sufficient NFκB activation for viral gene expression and suppressing proinflammatory responses derived from factors such as tetherin.

While tetherin activated only NFκB reporter genes in 293 cells, in some donors’ T cells, tetherin-sensitive HIV-1 also induced detectable type 1 interferon secretion. Again this was tetherin dependent, as shRNAs against tetherin abolished it. Since Vpu is not a constituent of incoming viral particles, this tetherin dependence of type 1 IFN induction indicates that it requires a productive round of viral replication, rather than direct sensing of virion components from the inoculum. The upregulation of IFNβ by Vpu(−) HIV-1 and HIV-1 Vpu-A14L, but not CXCL10, was reduced by inhibitory peptides against TRIF, strongly suggesting TLR3 as the source, which is known to be expressed on some T cell subsets (Holm et al., 2009). As TLR3 resides primarily in endosomes(Takeuchi and Akira, 2010), these data suggest that immobilization and endocytic uptake of virions by tetherin may also lead to augmented recognition of viral components by membrane-associated PRRs expressed in primary HIV-1 target cells (for example, virion-associated RNA molecules that may be liberated upon virion degradation). Low levels of activated IRF3 could be detected in all virus-infected T cells 48 hr after infection, contrary to a recent study that suggested IRF3 itself is degraded by Vpu (Doehle et al., 2012). While purified HIV-1 particles do not directly induce strong inflammatory responses in most target cells, a recent study has indicated that HIV-infected cells can trigger interferon release from bystanders through the activation of IRF3 (Lepelly et al., 2011). This appears to be related to cell-to-cell transmission of fusion-competent virions, something that could conceivably be further augmented in this case by tetherin-mediated virion retention (Jolly et al., 2010). Finally, tetherin’s identification as a regulator of TLR7 activity in plasmacytoid dendritic cells via the inhibitory leukocyte receptor ILT7 suggests that its signaling properties may have further roles in host-cell pattern recognition (Cao et al., 2009). The further characterization of virus-induced tetherin signaling in primary cells and the cellular factors involved will therefore determine the importance of this attribute in innate immunity.

Tetherin counteraction is conserved among human and primate lentiviruses, but the viral protein that performs this role varies (Le Tortorec et al., 2011). While tetherin restricts cell-free viral release, it poorly inhibits cell-to-cell viral transfer between CD4+ T cells (Casartelli et al., 2010; Jolly et al., 2010), which likely represents a major route of viral spread in vivo (Jolly and Sattentau, 2004). This suggests that avoidance of tetherin-mediated restriction is required for initial viral transmission, and/or tetherin’s antiviral activity imposes an additional inhibition of viral replication in vivo beyond simply blocking cell-free viral production. For example, accumulation of virions on cell surfaces by tetherin may promote recognition of infected cells through the deposition of antibodies and subsequent clearance by phagocytes. The acquisition of the coupling of tetherin activity to direct proinflammatory gene expression via NFκB activation may therefore significantly augment this by attracting other leukocytes to a site of infection. The threshold cellular levels of tetherin that can induce signaling may well be lower than those required for potent virion retention, providing further impetus for human viruses to efficiently remove tetherin from the PM. We note with interest that while the Nef and Env countermeasures of SIVs and HIV-2 do not promote tetherin degradation, HIV-1 Vpu does (Le Tortorec et al., 2011). Moreover, only Vpu proteins from the pandemic M Group of HIV-1 have acquired efficient tetherin counteraction (Sauter et al., 2009). Thus we speculate that the signaling activity of human tetherin that we have uncovered contributes to its role in the early innate response to HIV-1 and other viral infections, and may provide further selective pressure on enveloped viral pathogens to counteract its activity. The findings presented herein provide an intriguing example of a functional activity within a mediator of intrinsic viral defense that may have arisen to augment its overall potency in response to pressure by viral proteins that target its antiviral activity.

## Experimental Procedures

### Plasmids and Cells

The molecular clones of HIV-1 NL4.3 and derived Vpu mutants have been described previously (Neil et al., 2006; Vigan and Neil, 2010). Tetherin mutants and species orthologs were made in pCR3.1 by standard molecular biological methods. Chimpanzee tetherin was provided by G. Towers (UCL). pCR3.1-GFP-EBoV Vp40, pMLV NCS (MLV), pMLVp6py, and pMLV NCS Δpy plasmids were provided by J. Martin-Serrano (KCL) (Martin-Serrano et al., 2004). The NFκB-dependent 3xkB-pCONA-FLuc reporter construct, the CMV-RLuc control plasmid, and human TRAF6 cDNAs were provided by A. MacDonald (Mankouri et al., 2010) (University of Leeds). TRAF6 was subcloned with an N-terminal HA tag into pCR3.1. The MAVS expression vector was provided by J. Luban (University of Geneva) and the IFNβ-promoter luciferase vectors by M. Malim.

293 and 293T cells were obtained from the ATCC, while the reporter cell line HeLa-TZMbl was provided by John Kappes through the NIH AIDS Reagents Repository Program (ARRP). All adherent cells were maintained in DMEM supplemented with 10% fetal calf serum (FCS) and gentamicin. Derivatives stably expressing human tetherin or mutants thereof were produced by transducing the cells with MLV-based retroviral vectors packaging a pLHCX (Clontech) vector genome encoding the tetherin construct and selecting the cells in hygromycin (Invitrogen).

Peripheral blood mononuclear cells were isolated from freshly drawn whole blood on Lymphoprep (Axis-Shield). CD4+ T cells were then purified using an Untouched CD4+ T cell Dynabead kit (Invitrogen), and then activated for 48 hr with anti-CD3/anti-CD28 Dynabeads (Invitrogen) prior to use and cultured in RPMI/10% FCS/gentamicin and 30 U/ml recombinant IL-2. CD4 and tetherin expression were determined by flow cytometry using anti-CD4 APC (Becton Dickinson) or anti-human BST2-PE (eBiosciences). HIV-1 infection of T cells was monitored by flow cytometry using an intracellular anti-p24-PE (Beckam Coulter) staining.

### Reporter Gene Assays

For transient reporter gene assays, 10^5^ 293 cells were transfected with 50 ng of pCR3.1 tetherin plasmid or control pCR3.1 YFP in combination with 10 ng of 3xκB-pCONA-FLuc or IFNβ-luciferase reporter and 5 ng pCMV-RLuc. Forty-eight hours after transfection, firefly and Renilla luciferase activity in cell lysates was measured using a dual luciferase kit (Promega) and normalized. For antibody crosslinking experiments, 293 or 293THN cells were transfected with 10 ng of 3xκB-pCONA-FLuc and 5 ng pCMV-RLuc. Six hours later, the cells were treated with a 1:100 dilution of rabbit-anti-BST2 polyclonal Ab (Miyagi et al., 2009) (provided by K. Strebel through the NIH ARPP) followed by addition of a donkey anti-rabbit secondary. Cell lysates were taken at various time points thereafter and subjected to SDS-PAGE and western blot with rabbit anti-IκB-α (Cell Signaling), rabbit monoclonal anti-Phospho-IKKα/β (Ser176/180-clone 16A6, Cell Signaling), rabbit monoclonal anti-IRF3 (clone D83B9, Cell Signaling), rabbit monoclonal anti-Phospho-IRF3 (clone S396, Cell Signaling), rabbit anti-RelA, and rabbit anti-hsp90 (Santa Cruz Biotechnologies) and visualized either by Li-Cor apparatus using fluorophore-conjugated secondary antibody (IRDye 800 Goat anti-rabbi IRDye 680 Goat anti-mouse) or by ImageQuant using anti-rabbit HRP-linked secondary antibodies (NEB, UK). Luciferase activities were determined at 24 hr posttreatment.

For viral-mediated reporter gene assays, 293 or 293THN cells were transfected with 10 ng of 3xkB-pCONA-FLuc (or IFNβ luciferase reporter) and 5 ng pCMV-RLuc in combination with 500 ng pNL4.3, pNL4.3 Vpu mutant, pCR3.1-GFP-EBoV VP40), MLV, MLVΔPY, or MLVΔPY/p6 (Martin-Serrano et al., 2001. For experiments with MLV or derivatives, 200 ng of pCNCG and 100 ng of pVSV-G were cotransfected. Luciferase activities were measured 48 hr posttransfection. Infectivity of viral supernatants and biochemical analysis of cell lysates and viral particles were performed as described below in “Virus Stocks and Release Assays.”

For transient knockdown of signaling components, 105 cells were seeded per well of a 24-well plate, and 6 hr later the cells were transfected with 50 pmol of siRNA in complex with Dharmofect-1 (Dharmacon). Forty-eight hours later, cells were reseeded and 6 hr later cotransfected with a second dose of siRNA alongside reporter constructs as per the transient reporter gene assays. The following siRNAs were used: from QIAGEN, MAP3K7_5 (TAK1) (SI00300741); TRAF2_7 (SI03096009) for TRAF2; TRAF6_7 and _8 (SI03046043 and SI03050145) for TRAF6; Dharmacon ON-TARGETplus SMARTpools for human AP2M1 (L-008170-00-0005) for AP2 and human UBE2N (L-003920-00-0005) for Ubc13 and siControl nontargeting pool (D-001810-10-20) as the siRNA control. Protein knockdowns were verified by western blot using the following antibodies: rabbit anti-TAK1, rabbit anti-TRAF2 (c192), rabbit anti-Ubc13 (Cell Signaling Technology), rabbit anti-TRAF6 (H274, Santa Cruz), and mouse anti-AP2μ1 (BD Biosciences).

### Virus Stocks and Release Assays

HIV-1 NL4.3 and Vpu mutant viral stocks were produced in 293T cells by transient transfection, and endpoint titers were determined on HeLa-TZM indicator cells as described previously (Le Tortorec and Neil, 2009). For primary cell infections, viral stocks were treated for 2 hr with 10 U/ml DNase-I (Roche) and then concentrated by ultracentrifugation through a 20% sucrose/PBS cushion (30,000 rpm on a Sorvall SW41 rotor for 90 min) and resuspended in RPMI.

For viral release assays, CD4+ T cells were infected with HIV-1 NL4.3 or Vpu mutant at the required multiplicity, and washed. Forty-eight hours later, cell lysates and filtered viral supernatants were harvested. The infectivity of viral supernatants was determined on HeLa-TZMbl cells as described (Le Tortorec and Neil, 2009). MLV titers were determined by transducing 293T cells and enumerating the GFP-positive cells by FACS 48 hr later. HIV-1 particle release was determined by quantitative western blot, as described (Vigan and Neil, 2010).

### Primary Cell Infections

5 × 10^5^ activated CD4+ T cells were infected at a multiplicity of infection (moi) of 5 to ensure >90% p24+ cells 48 hr postinfection. Cells and supernatants were then harvested and assayed for viral gene expression, and virus particle production as described above. Cells were also harvested for total RNA and supernatants for cytokine production. When required, equal amounts of activated CD4+ T cells were transduced with lentiviral vectors (pCSGW) encoding U6-driven shRNA-GFP, 5′-GUUCAUCUGCACCACCGGCAAGCUUCGGCUUGCCGGUGGUGCAGAUACUU-3′; or shRNA-THN, 5′-GGAGTTCTGGTGTTCCTGATTATTTCGATGATCAGGAGCACCGAATTCC-3′, provided by G. Towers (UCL) at an input equivalent to a moi of 5 when titered on 293T cells. The transduced cells were infected 24 hr later and harvested as described above. Surface tetherin levels were determined by flow cytometry at 48 hr posttransduction. For TLR inhibition studies, infected cells were cultured in the presence of 40 μM of control, TRIF, and MyD88-inhibitory peptides (Invivogen).

### Quantitative RT-PCR

Total RNA was isolated and purified from transfected or infected cells using a QIAGEN RNeasy kit, and 50 ng of RNA was reverse transcribed by random hexamer priming using a High Capacity cDNA Reverse Transcription kit (ABI). Of the reaction, 5 μl was subjected to quantitative PCR using ABI primer/probe sets for human *Cxcl10*, *IL6*, *Ifnb*, and *Gapdh* in an ABIPrism 7900HT Sequence Detections System. Ct data was processed relative to the GAPDH control using the ABI RQ Manager 1.2 software.

### Cytokine Production

Supernatants for infected T cells were analyzed for CXCL10 and IL-6 protein levels using specific Quantikine ELISAs (R+D Systems). Levels of bioactive type-I IFN were determined using HEK-Blue indicator cells (InVivoGen).

### Immunoprecipitations

293 cells were transfected with 700 ng of HA-TRAF6 in combination with equal quantities of pCR3.1-GFP or pCR3.1 tetherin constructs. Forty-eight hours posttransfection, cells were lysed in 1% digitonin in 50 mM Tris-HCL (pH 7.4)/150 mM NaCl with protease inhibitors (Roche) and N-ethymaleimide. Postnuclear supernatants were immunoprecipitated with 5 μg of anti-HA.11 mAb (Covance) and protein G agarose beads (Invitrogen). Cell lysates and IPs were subjected to SDS-PAGE and western blots performed using rabbit anti-HA (Rockland) and rabbit anti-BST2 antibodies and visualized by ImageQuant using HRP-linked anti-rabbit secondary Abs (NEB, UK).

### Ethics Statement

Ethical approval for the drawing of blood and preparation of leukocyte subsets from healthy donors following written informed consent was obtained through the King’s College London Infectious Disease BioBank Local Research Ethics Committee (under the authority of the Southampton and South West Hampshire Research Ethics Committee—approval REC09/H0504/39), approval number SN-1/6/7/9.

## References

[bib1] Agromayor M., Soler N., Caballe A., Kueck T., Freund S.M., Allen M.D., Bycroft M., Perisic O., Ye Y., McDonald B. (2012). The UBAP1 subunit of ESCRT-I interacts with ubiquitin via a SOUBA domain. Structure.

[bib2] Cao W., Bover L., Cho M., Wen X., Hanabuchi S., Bao M., Rosen D.B., Wang Y.H., Shaw J.L., Du Q. (2009). Regulation of TLR7/9 responses in plasmacytoid dendritic cells by BST2 and ILT7 receptor interaction. J. Exp. Med..

[bib3] Casartelli N., Sourisseau M., Feldmann J., Guivel-Benhassine F., Mallet A., Marcelin A.G., Guatelli J., Schwartz O. (2010). Tetherin restricts productive HIV-1 cell-to-cell transmission. PLoS Pathog..

[bib4] Doehle B.P., Chang K., Rustagi A., McNevin J., McElrath M.J., Gale M. (2012). Vpu mediates depletion of interferon regulatory factor 3 during HIV infection by a lysosome-dependent mechanism. J. Virol..

[bib5] Dubé M., Paquay C., Roy B.B., Bego M.G., Mercier J., Cohen E.A. (2011). HIV-1 Vpu antagonizes BST-2 by interfering mainly with the trafficking of newly synthesized BST-2 to the cell surface. Traffic.

[bib6] Gupta R.K., Hué S., Schaller T., Verschoor E., Pillay D., Towers G.J. (2009). Mutation of a single residue renders human tetherin resistant to HIV-1 Vpu-mediated depletion. PLoS Pathog..

[bib7] Holm C.K., Petersen C.C., Hvid M., Petersen L., Paludan S.R., Deleuran B., Hokland M. (2009). TLR3 ligand polyinosinic:polycytidylic acid induces IL-17A and IL-21 synthesis in human Th cells. J. Immunol..

[bib8] Janvier K., Pelchen-Matthews A., Renaud J.B., Caillet M., Marsh M., Berlioz-Torrent C. (2011). The ESCRT-0 component HRS is required for HIV-1 Vpu-mediated BST-2/tetherin down-regulation. PLoS Pathog..

[bib9] Jia B., Serra-Moreno R., Neidermyer W., Rahmberg A., Mackey J., Fofana I.B., Johnson W.E., Westmoreland S., Evans D.T. (2009). Species-specific activity of SIV Nef and HIV-1 Vpu in overcoming restriction by tetherin/BST2. PLoS Pathog..

[bib10] Jolly C., Sattentau Q.J. (2004). Retroviral spread by induction of virological synapses. Traffic.

[bib11] Jolly C., Booth N.J., Neil S.J. (2010). Cell-cell spread of human immunodeficiency virus type 1 overcomes tetherin/BST-2-mediated restriction in T cells. J. Virol..

[bib12] Jouvenet N., Neil S.J., Zhadina M., Zang T., Kratovac Z., Lee Y., McNatt M., Hatziioannou T., Bieniasz P.D. (2009). Broad-spectrum inhibition of retroviral and filoviral particle release by tetherin. J. Virol..

[bib13] Kueck T., Neil S.J. (2012). A cytoplasmic tail determinant in HIV-1 Vpu mediates targeting of tetherin for endosomal degradation and counteracts interferon-induced restriction. PLoS Pathog..

[bib14] Lau D., Kwan W., Guatelli J. (2011). Role of the endocytic pathway in the counteraction of BST-2 by human lentiviral pathogens. J. Virol..

[bib15] Le Tortorec A., Neil S.J. (2009). Antagonism to and intracellular sequestration of human tetherin by the human immunodeficiency virus type 2 envelope glycoprotein. J. Virol..

[bib16] Lepelly A., Louis S., Sourriseau M., Law H.K., Pothlichet J., Schilte C., Chaperot L., Plumas J., Randall R.E., Si-Tahar M. (2011). Innate sensing of HIV-infected cells. PLoS Pathog..

[bib17] Le Tortorec A., Willey S., Neil S.J. (2011). Antiviral inhibition of enveloped virus release by tetherin/BST-2: action and counteraction. Viruses.

[bib18] Liberatore R.A., Bieniasz P.D. (2011). Tetherin is a key effector of the antiretroviral activity of type I interferon in vitro and in vivo. Proc. Natl. Acad. Sci. USA.

[bib19] Lim E.S., Malik H.S., Emerman M. (2010). Ancient adaptive evolution of tetherin shaped the functions of Vpu and Nef in human immunodeficiency virus and primate lentiviruses. J. Virol..

[bib20] Mankouri J., Fragkoudis R., Richards K.H., Wetherill L.F., Harris M., Kohl A., Elliott R.M., Macdonald A. (2010). Optineurin negatively regulates the induction of IFNbeta in response to RNA virus infection. PLoS Pathog..

[bib21] Martin-Serrano J., Neil S.J. (2011). Host factors involved in retroviral budding and release. Nat. Rev. Microbiol..

[bib22] Martin-Serrano J., Zang T., Bieniasz P.D. (2001). HIV-1 and Ebola virus encode small peptide motifs that recruit Tsg101 to sites of particle assembly to facilitate egress. Nat. Med..

[bib23] Martin-Serrano J., Perez-Caballero D., Bieniasz P.D. (2004). Context-dependent effects of L domains and ubiquitination on viral budding. J. Virol..

[bib24] Matsuda A., Suzuki Y., Honda G., Muramatsu S., Matsuzaki O., Nagano Y., Doi T., Shimotohno K., Harada T., Nishida E. (2003). Large-scale identification and characterization of human genes that activate NF-kappaB and MAPK signaling pathways. Oncogene.

[bib25] McNatt M.W., Zang T., Hatziioannou T., Bartlett M., Fofana I.B., Johnson W.E., Neil S.J., Bieniasz P.D. (2009). Species-specific activity of HIV-1 Vpu and positive selection of tetherin transmembrane domain variants. PLoS Pathog..

[bib26] Miyagi E., Andrew A.J., Kao S., Strebel K. (2009). Vpu enhances HIV-1 virus release in the absence of Bst-2 cell surface down-modulation and intracellular depletion. Proc. Natl. Acad. Sci. USA.

[bib27] Neil S.J., Eastman S.W., Jouvenet N., Bieniasz P.D. (2006). HIV-1 Vpu promotes release and prevents endocytosis of nascent retrovirus particles from the plasma membrane. PLoS Pathog..

[bib28] Neil S.J., Sandrin V., Sundquist W.I., Bieniasz P.D. (2007). An interferon-alpha-induced tethering mechanism inhibits HIV-1 and Ebola virus particle release but is counteracted by the HIV-1 Vpu protein. Cell Host Microbe.

[bib29] Neil S.J., Zang T., Bieniasz P.D. (2008). Tetherin inhibits retrovirus release and is antagonized by HIV-1 Vpu. Nature.

[bib30] Perez-Caballero D., Zang T., Ebrahimi A., McNatt M.W., Gregory D.A., Johnson M.C., Bieniasz P.D. (2009). Tetherin inhibits HIV-1 release by directly tethering virions to cells. Cell.

[bib31] Pertel T., Hausmann S., Morger D., Züger S., Guerra J., Lascano J., Reinhard C., Santoni F.A., Uchil P.D., Chatel L. (2011). TRIM5 is an innate immune sensor for the retrovirus capsid lattice. Nature.

[bib32] Postler T.S., Desrosiers R.C. (2012). The cytoplasmic domain of the HIV-1 glycoprotein gp41 induces NF-κB activation through TGF-β-activated kinase 1. Cell Host Microbe.

[bib33] Rollason R., Korolchuk V., Hamilton C., Schu P., Banting G. (2007). Clathrin-mediated endocytosis of a lipid-raft-associated protein is mediated through a dual tyrosine motif. J. Cell Sci..

[bib34] Sauter D., Schindler M., Specht A., Landford W.N., Münch J., Kim K.A., Votteler J., Schubert U., Bibollet-Ruche F., Keele B.F. (2009). Tetherin-driven adaptation of Vpu and Nef function and the evolution of pandemic and nonpandemic HIV-1 strains. Cell Host Microbe.

[bib35] Schmidt S., Fritz J.V., Bitzegeio J., Fackler O.T., Keppler O.T. (2011). HIV-1 Vpu blocks recycling and biosynthetic transport of the intrinsic immunity factor CD317/tetherin to overcome the virion release restriction. MBio.

[bib36] Serra-Moreno R., Jia B., Breed M., Alvarez X., Evans D.T. (2011). Compensatory changes in the cytoplasmic tail of gp41 confer resistance to tetherin/BST-2 in a pathogenic nef-deleted SIV. Cell Host Microbe.

[bib37] Skaug B., Jiang X., Chen Z.J. (2009). The role of ubiquitin in NF-kappaB regulatory pathways. Annu. Rev. Biochem..

[bib38] Takeuchi O., Akira S. (2010). Pattern recognition receptors and inflammation. Cell.

[bib39] Tokarev A.A., Munguia J., Guatelli J.C. (2011). Serine-threonine ubiquitination mediates downregulation of BST-2/tetherin and relief of restricted virion release by HIV-1 Vpu. J. Virol..

[bib40] Vigan R., Neil S.J. (2010). Determinants of tetherin antagonism in the transmembrane domain of the human immunodeficiency virus type 1 Vpu protein. J. Virol..

[bib41] Zhang F., Wilson S.J., Landford W.C., Virgen B., Gregory D., Johnson M.C., Munch J., Kirchhoff F., Bieniasz P.D., Hatziioannou T. (2009). Nef proteins from simian immunodeficiency viruses are tetherin antagonists. Cell Host Microbe.

