# Supplementary material for: Innate Sensing of HIV-1 Assembly by Tetherin Induces NFκB-Dependent Proinflammatory Responses
Source: Cell Host Microbe. 2012 Nov 15;12(5):633–44. doi: 10.1016/j.chom.2012.10.007 (PMC3556742; doi:10.1016/j.chom.2012.10.007)
Supplement: Document S1. Figure S1, Figure S2, Figure S3, Figure S4, Figure S5 [file mmc1.pdf]

Cell Host & Microbe, Volume *12*

Supplemental Information

Innate Sensing of HIV-1 Assembly by Tetherin Induces NFκB-Dependent Proinflammatory Responses

Rui Pedro Galão, Anna Le Tortorec, Suzanne Pickering, Tonya Kueck, and Stuart J.D. Neil

Supplementary Figure Inventory

Figure S1: Control Panels demonstrating parallel virus release from experiments in **Figure 1.**

Figure S2: Controls in cell lines and primary cells to demonstrate the potential source of IFN responses observed in **Figure 2** and the specificity of shRNA constructs**.**

Figure S3 Further virological and signaling phenotypes of tetherin mutants described in **Figure 3**

Figure S4: Interactions of some of the tetherins from **Figures 5** with TRAF6

Figure S5: Virological phenotypes, expression levels and TRAF6 interactions of primate tetherins and chimeric proteins from **Figure 6.**

Supplementary Figures

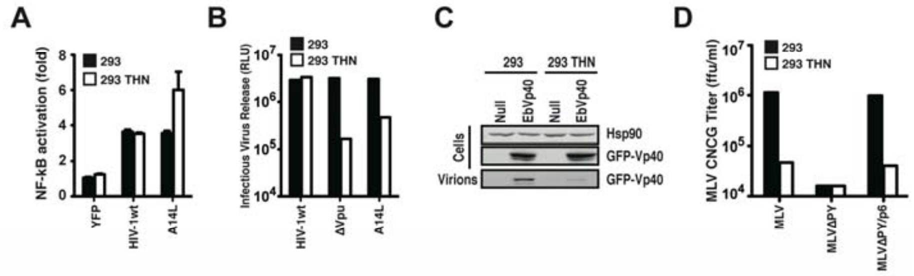

Figure S1, related to Figure 1: Restriction of viral particle release in 293THN cells. (A) Transfection of an HIV-1 Vpu-A14L provirus enhanced NF-kB reporter gene activation in 293THN cells. Data are represented as mean +/- SEM of 3 independent experiments **(B)** Parallel infectious virus release from **(A)** and Figure 1D on HeLa-TZM indicator cells. **(C)** Ebola virus-like particles released from 293 and 293THN cells from Figure 1E were analyzed by western blotting of cell lysates and pelleted supernatants. **(D)** MLV infectious virus release by titration of supernatants from Figure 1G on 293T and analyzed by flow cytometry 48h later.

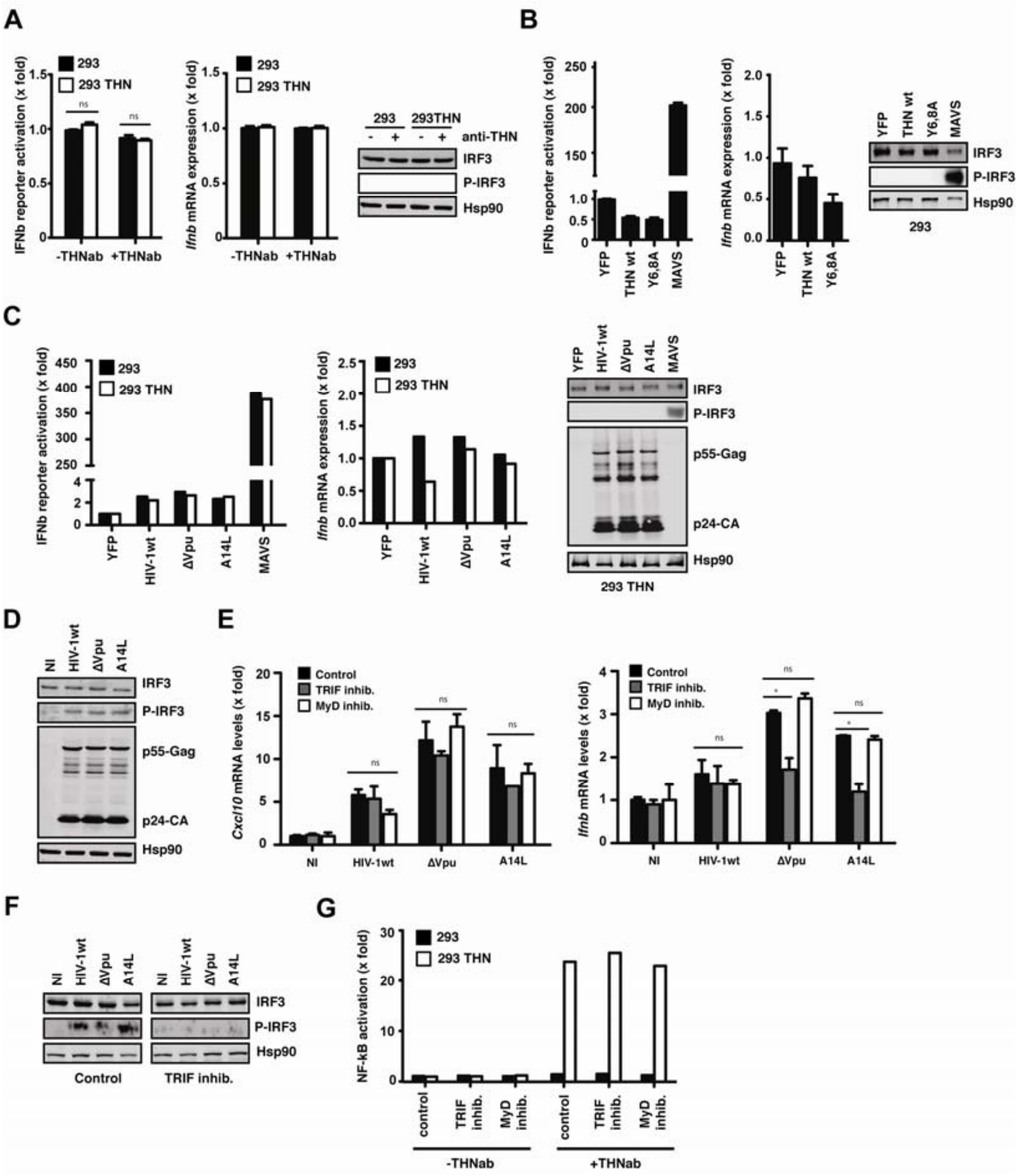

Figure S2, related to Figure 2: Tetherin does not directly activate IFN□ expression and IRF3 phosphorylation, but does so indirectly through a TRIF-dependent mechanism in infected T cells. IFNß-luciferase reporter activation (left panels), induction of IFNß mRNA (central panels) or phosphorylation of IRF3 (right panels) are not observed following crosslinking with a rabbit anti-tetherin antibody **(A)**, tetherin overexpression **(B)** or Vpu(-)

HIV-1 assembly **(C)**. **(D)** Purified CD4+ T cells were infected with the indicated virus at an MOI of 5. 48h later IRF-3 phosphorylation and viral infection was analyzed by western blot of cell lysates. **(E)** Infected CD4+ T cells were cultured in the presence of 40μM of control, TRIF and MyD88 inhibitory peptides. Total RNA from 3 biological replicates of parallel cultures were analyzed for *Cxcl10*, and *Ifnb* mRNA levels relative to *Gapdh* by qRT-PCR. Fold changes relative to non-infected cells treated with control peptide. * P>0.05, ** P>0.01 and *** P>0.001 as determined by two-tailed ttest. **(F)** Activation state of IRF3 in cells cultured in the presence of TRIF inhibitory peptides was assessed by western blotting cell lysates from (E) . **(G)** Activation of NFkB signaling by anti-tetherin antibody crosslink in 293THN cells is not affected by TRIF or MyD88 inhibitory peptides. **(H)** CD4+ T cells were transduced with lentiviral vectors encoding shRNAs against GFP (shcontrol) or tetherin (sh-THN) and infected with indicated virus at MOI 5 as in Figure 2E. Cell surface staining for endogenous tetherin was analyzed by flow cytometry 48h post-transduction. The solid peak represents the binding of the isotype control and the lines represent tetherin levels in non-treated cells (NT, dotted lines) or transduced cells (solid lines). **(I)** Parallel infectious virus release from **(H)** on HeLa-TZM indicator cells. All error bars represent means +/- SEM of 3 independent experiments.

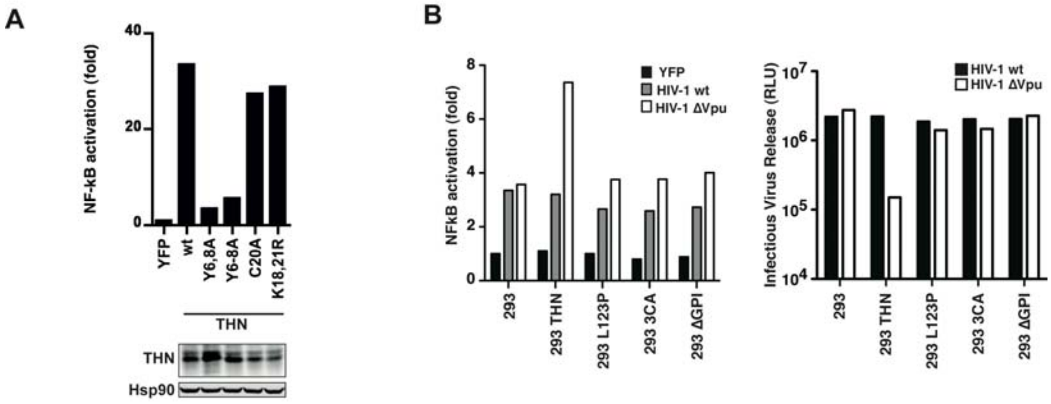

Figure S3, related to Figure 3: NF-kB reporter gene activities and antiviral function of tetherin mutants. (A) NF-kB reporter gene activities of tyrosine, cysteine and membrane proximal ubiquitin-accepting-lysine residue mutant tetherins determined by transient transfection as in Figure 3A. **(B)** NF-kB reporter gene activity in 293 cells expressing the indicated tetherin extracellular mutant with wildtype or Vpu(-) provirus as in Figure 3B, and parallel infectious virus release determined on HeLa-TZM cells (right panel).

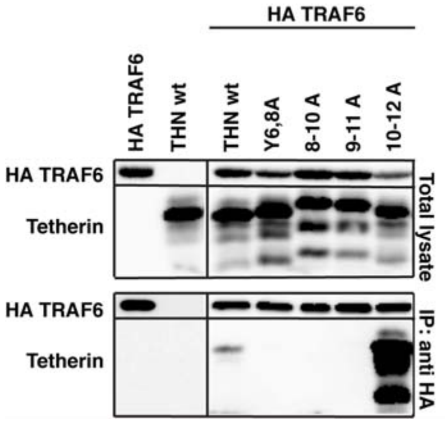

Figure S4, related to Figure 5: Co-IP of further tetherin mutants with TRAF6. Co-immunoprecipitation of tetherin, tetherin cytoplasmic tail mutants with HA-TRAF6 from transiently transfected 293 cells as in Figure 5C.

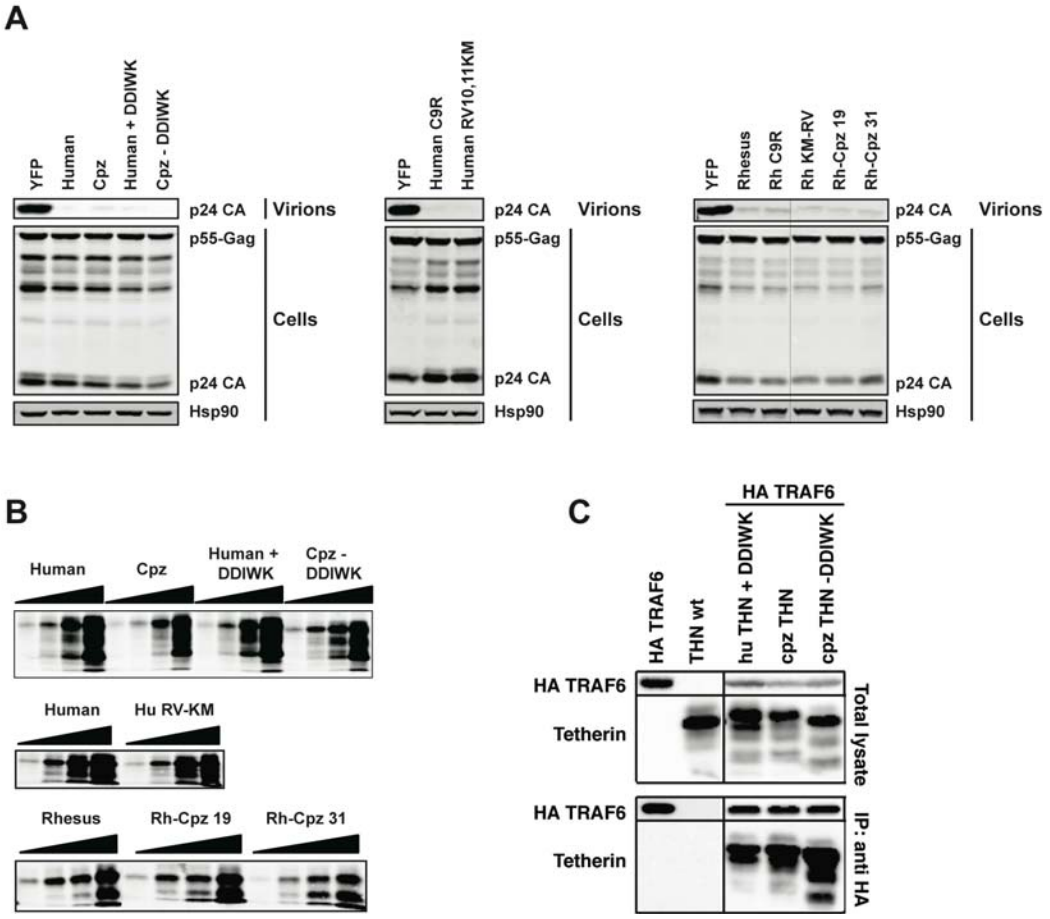

Figure S5, related to Figure 6: Antiviral function of species-specific tetherin mutants. Tetherins from Figure 6 were tested for their ability to restrict Vpu(-) virus release (A) and protein expression (B). (C) Chimpanzee and human tetherins with or without DDIWK motif were assessed for their ability to be co-precipitated with HA-TRAF6. Control blot of single transfectants reproduced from Figure S4.
